# Isolation of pancreatic microbiota from cystic precursors of pancreatic cancer with intracellular growth and DNA damaging properties

**DOI:** 10.1080/19490976.2021.1983101

**Published:** 2021-11-24

**Authors:** Asif Halimi, Giorgio Gabarrini, Michał Jacek Sobkowiak, Zeeshan Ateeb, Haleh Davanian, Rogier Aäron Gaiser, Urban Arnelo, Roberto Valente, Alicia Y.W. Wong, Carlos Fernández Moro, Marco Del Chiaro, Volkan Özenci, Margaret Sällberg Chen

**Affiliations:** a Division of Surgery, Department of Clinical Science, Intervention and Technology, Karolinska Institutet, Stockholm, Sweden; b Department of Surgical and Perioperative Sciences, Surgery, Umeå University, Umeå, Sweden; c Department of Dental Medicine, Karolinska Institutet, Huddinge, Sweden; d Division of Clinical Microbiology, Department of Laboratory Medicine, Karolinska Institutet, Huddinge, Sweden; e Division of Pathology, Department of Laboratory Medicine, Karolinska Institutet, Huddinge, Sweden; f Department of Clinical Pathology/Cytology, Karolinska University Hospital, Huddinge, Sweden; g Department of Surgery, University of Colorado Denver – Anschutz Medical Campus, Aurora, CO, USA; h Department of Clinical Microbiology F 72, Karolinska University Hospital, Huddinge, Stockholm, Sweden

**Keywords:** Pancreatic cancer, pancreatic cystic neoplasm, IPMN, microbiota

## Abstract

Emerging research suggests gut microbiome may play a role in pancreatic cancer initiation and progression, but cultivation of the cancer microbiome remains challenging. This pilot study aims to investigate the possibility to cultivate pancreatic microbiome from pancreatic cystic lesions associated with invasive cancer. Intra-operatively acquired pancreatic cyst fluid samples showed culture-positivity mainly in the intraductal papillary mucinous neoplasm (IPMN) group of lesions. MALDI-TOF MS profiling analysis shows Gammaproteobacteria and Bacilli dominate among individual bacteria isolates. Among cultivated bacteria, Gammaproteobacteria, particularly *Klebsiella pneumoniae*, but also *Granulicatella adiacens* and *Enterococcus faecalis*, demonstrate consistent pathogenic properties in pancreatic cell lines tested in *ex vivo* co-culture models. Pathogenic properties include intracellular survival capability, cell death induction, or causing DNA double-strand breaks in the surviving cells resembling genotoxic effects. This study provides new insights into the role of the pancreatic microbiota in the intriguing link between pancreatic cystic lesions and cancer.

## Introduction

Pancreatic cancer (PC) is one of the most aggressive and lethal types of cancer. The 5-year survival rate at the time of diagnosis is about 10%, as approximately 80–85% of patients present either unresectable or metastatic disease.^1^ PC accounts for roughly 459 000 new cases and 432 000 deaths according to GLOBOCAN 2018 estimates.^1^ Emerging global data indicate two to three-fold increase in the diagnosis and mortality of PC, especially in countries with higher social-demographic indices. It is predicted that PC will soon surpass breast cancer as the third leading cause of cancer-related death in the European Union.^2^ Most PCs are characterized as ductal adenocarcinoma (PDAC) and represent malignancy of the exocrine pancreas. One of major risk factors for PDAC is pancreatic cystic neoplasms (PCNs), especially the mucinous group of tumors. As the use of high-quality cross-sectional imaging increases, PCNs are now reported in up to 49% of MRI tested individuals, with intraductal papillary mucinous neoplasms (IPMNs) being the most common.^3–5^ Individuals with IPMNs are at increased risk (1–8%) of developing conventional PDAC elsewhere in the pancreas and IPMNs tend to co-localize with PDAC with a distinct genetic signature.^6^ IPMNs are therefore considered premalignant and require either surveillance or surgical resection due to the risk of malignant transformation.^7^ Pathologically, IPMNs are distinguished as exhibiting low-grade dysplasia (LGD) or high-grade dysplasia (HGD). Despite improved diagnosis and management of PCNs, the pre-operative differentiation between the various types of PCN and for neoplastic grading is still a significant clinical challenge.^5^

Emerging reports in the last years indicate that the pancreas, an organ previously thought to be sterile, appears to harbor a unique microbiome. Recent studies^8,9^ in experimental animal models further showed that the bacteria derived from the pancreatic microbiome can metabolize cancer drugs, rendering the cancer chemotherapy less efficient, and drive immunosuppression and oncogenesis. Moreover, tumor microbiome diversity and composition appear to have predictive value for patient survival via a mechanism thought to involve immunity within the tumor environment.^10^ In line with this, we recently reported that cyst fluid in IPMNs harbors a distinctive tumor microbiome signature.^11^ This microbiome signature involves inflammation and microbial translocation components, as shown by protein and metabolome measurements in the aspirated cyst fluid from surgically retrieved IPMNs, indicated by our previous cohort studies.^11–13^

Clearly, these recent insights underline the importance of microbiome analysis as an alternative approach to further improve clinical diagnosis and treatment regiments for pancreatic disease.^14^ Nevertheless, most of the current understanding of the pancreatic microbiome is acquired through microbial gene analysis and not by functional examination on live bacteria. Whether human pancreas-derived bacteria may cause direct cellular injury remains largely unexplored. We report here a first pilot study investigating the culturable pancreatic in microbiota a cohort of PCN patients with cancer suspicion who underwent surgery.

Cultures of peri-operatively aspirated PCN fluid with the majority belonging to cases verified as IPMN with HGD or IPMN associated with invasive cancer showed mainly polymicrobial growth, i.e. presence of more than one bacterial species. Subsequent co-culture experiments in three pancreatic cell lines with various cancer mutation complexity revealed several types of pancreas cell insult including capability of intracellular survival, induction of cell death, and DNA double-strand breaks resembling genotoxic responses. This *in vitro* effect is significantly reduced by antibiotic treatment.

## Results

### Patient characteristics and culture positivity

Between February 2018 and November 2019, patients undergoing pancreatic surgery due to PCN with cancer suspicion confirmed by radiological and clinical examinations participated in this study. Surgically removed pancreata were sampled for cyst fluid immediately upon resection in strict sterile conditions and cultured in aerobic and anaerobic blood culture bottles. As shown in Tables 1, 7 of 29 cases (24%) exhibited bacterial growth. Compiled baseline characteristics of the culture-positive vs culture-negative groups indicate that risk factors associated with culture-positivity include higher patient age, elevated CRP, and history of invasive endoscopy, i.e. endoscopic retrograde cholangiopancreatography (ERCP), percutaneous transhepatic cholangiography (PTC), or endoscopic ultrasound (EUS) with puncture (*p* < .01). No difference was seen between the groups over gender but within both groups there were more females than males. Lower serum albumin was also noted in this group (*p* < .05). Final pancreas pathology reports revealed that all seven culture-positive cases had IPMN, of which five were in the HGD stage or associated with invasive cancer (5 of 7; 71.4%). The culture-negative cases included only six malignant cases (6/21; 27.3%), the others were low-risk tumors such as IPMN-LGD and serous cystic tumors (SCNs), three cases also had signs of concomitant pancreatitis. No significant difference was found on the lesion size assessed either by radiology or histopathology analysis.

**Table 1.** Patient cohort clinical characteristics classified by microbiological culture result

Parameters	Culture positive (n = 7)	Culture neg (n = 22)	*P*-value
***Demographics***			
**Age (years)**	78.0 ± 4.2	66.4 ± 13.1	**0.014**
**Male:Female**	1:6	7:15	0.30
**BMI**	24.8 ± 4.2	26.7 ± 5.1	0.40
***Blood tests prior surgery***			
**S-Ca19-9 (kE/L)**	142.6 ± 251.3	41.2 ± 50.3	0.31
**HbA1c (mmol/mol)**	46.6 ± 7.1	42.3 ± 9.1	0.23
**Serum amylase (microkat/L)**	0.74 ± 0.6	0.6 ± 0.8	0.39
**Albumin (g/L)**	29.9 ± 8.3	38.2 ± 2.5	**0.002**
**Bilirubin (micromol/L)**	11.1 ± 7.0	7.1 ± 2.6	0.18
**CRP**	27.0 ± 44.6	2.3 ± 1.9	**0.007**
**White blood cells (x10(9)/L)**	6.5 ± 3.3	7.5 ± 1.5	0.47
***Pancreas pathology***			
HGD or Cancer (%)*	71.4	27.3	0.07
IPMN:MCN:Others (%)*	100:0:0	64:5:32	n.d.
Lesion diameter (Pre-Op, mm)**	24.3 ± 10	38.8 ± 24.7	0.18
Lesion diameter (mm)*	22 ± 14	46–9 ± 34.6^#^	0.052
**Invasive endoscopy (ERCP, PTC, EUS with puncture) (YES, %)**	71.4	9.1	**0.004**
**Antibiotics 30 days prior to surgery (YES, %)**	28.6	4.5	0.09
***Pancreas microbial culture***			
**Polymicrobial (%)**	71	0	**0.0002**

Descriptive data are expressed as n (%) or mean ± standard deviation.

Mann-Whitney U test was used for analysis of quantitative datasets. Fisher´s exact test for nominal datasets.

*P*-values <0.05 were considered significant (indicated in bold).

*Determined by histopathological examination after operation.

**Determined by pre-operation radiology, diameter of pancreatic cyst or dilated main pancreatic duct.

# n = 21

Among the seven culture-positive cases, polymicrobial growth was found in five (71%). Notably, all patients had received standard antibiotic prophylaxis on the day of surgery with metronidazole and trimethoprim sulphamethoxazole. Treatment with antibiotics during the preceding month was also noted in some cases in both groups, without a significant group difference.

### Microbial cultivation and profiling

The culture-positive samples were streaked repetitively to obtain pure monocultures for a subsequent strain identification by MALDI-TOF MS profiling. As shown in Table 2, a total of 15 bacterial strains were identified, mainly facultative anaerobes of the class Gammaproteobacteria or Bacilli. Several *Klebsiella* spp., and *Enterococcus faecalis* and *Enterobacter cloacae* were also repeatedly noted in varying IPMN neoplastic grades: LGD (L) and HGD (H) or invasive cancer (C). Antibiotic susceptibility reports showed that the isolates were in general susceptible to antibiotics tested (data not shown). Bacteremia was detected in only two patients, both of which belonged to the IPMN-LGD group and showed no sign of bacterial growth in their cyst fluid (data not shown).

**Table 2.** Identity of pancreatic bacteria isolates performed by MALDI-TOF profiling and tissue pathology diagnosis

Sample ID	Intracystic isolate	Anaerobe	Gram	Phylum/class	Previous cancer association	Tissue diagnosis
L1	*Klebsiella aerogenes*	facult	N	Proteobacteria/Gammaproteobacteria	-	IPMN LGD
L2	*Enterococcus faecalis*	facult	P	Firmicutes/Bacilli	^15^	IPMN LGD
	*Enterobacter cloacae*	facult	N	Proteobacteria/Gammaproteobacteria	-	
H1	*Klebsiella oxytoca*	facult	N	Proteobacteria/Gammaproteobacteria	^16^	IPMN HGD
	*Granulicatella adiacens*	facult	P	Firmicutes/Bacilli	^11,17–21^	
	*Citrobacter freundii*	facult	N	Proteobacteria/Gammaproteobacteria	-	
H2	*Enterobacter cloacae*	facult	N	Proteobacteria/Gammaproteobacteria	-	IPMN HGD
	*Enterococcus faecium*	facult	P	Firmicutes/Bacilli	-	
	*Streptococcus anginosus* (milleri) group	facult	P	Firmicutes/Bacilli	^22,23^	
H3	*Streptococcus oralis*	facult	P	Firmicutes/Bacilli	-	IPMN HGD
C1	*Enterococcus faecalis*	facult	P	Firmicutes/Bacilli	^15^	IPMN + Cancer
	*Stenotrophomonas maltophilia*	no	N	Proteobacteria/Gammaproteobacteria	-	
C2	*Klebsiella pneumoniae*	facult	N	Proteobacteria/Gammaproteobacteria	^24^	IPMN + Cancer
	*Streptococcus anginosus* (milleri) group	facult	P	Firmicutes/Bacilli	^22,23^	
	*Enterobacter cloacae*	facult	N	Proteobacteria/Gammaproteobacteria	-	

### Bacteria from IPMN cyst fluid can survive intracellularly in healthy pancreatic cells and PDAC cells

As bacterial adherence to epithelial cells and intracellular survival are important virulence factors, we investigated whether the bacterial isolates from pancreatic cyst fluid possessed these abilities. To address this question, cell line models representing healthy pancreatic cells (hTERT-HPNE) or PC cells of early (Capan-2) and late (AsPC-1) differentiation stage were co-incubated with individual bacterial strains for two hours and tested in a subsequent Gentamycin Protection Assay (GPA). The assay indicated that this short co-culture with pancreatic cells allowed most of the bacterial isolates to enter and survive inside the human pancreatic cells (Table 3). Among the top superior survivors noted in the healthy pancreatic cells were *Enterobacter cloacae* H2 (HGD 2), *Enterococcus faecium* H2, *Enterococcus faecalis* L2 (LGD 2) and *Klebsiella pneumoniae* C2 (Cancer 2). Those strains also survived well in both cancer cell types, especially *E. cloacae* H2, *E. faecium* H2 and *E. faecalis* L2. In the controls with bacteria alone without pancreas cell co-culture, gentamycin had complete (100%) bactericidal effect and no live bacteria were detected by the GPA assay. Our results thus indicate that bacteria from IPMN cyst fluid can invade and survive intracellularly in pancreatic cells *in vitro*, representing a potential reservoir and microbial mechanism to persist in both healthy and cancerous pancreatic tissues.

**Table 3.** DNA damage caused by bacterial infection and intracellular survival in healthy (hTERT-HPNE), early (Capan-2) and late-stage differentiation cancer (AsPC-1) cell lines

Sample ID	Isolate	hTERT-HPNE			Capan-2			AsPC-1		
		%pH2A.X+	%Cell death	%Intracellular survival	%pH2A.X+	%Cell death	%Intracellular survival	%pH2A.X+	%Cell death	%Intracellular survival
L1	*K. aerogenes*	36.70 ± 5.51	45.40 ± 3.32***	0.92 ± 0.17	74.77 ± 8.63*	52.27 ± 1.16***	0.20 ± 0.06	33.07 ± 7.52	45.57 ± 1.96*	0.48 ± 0.12
L2	*E. cloacae*	45.17 ± 8.80*	38.00 ± 11.27*	0.01 ± 0.002	66.77 ± 7.67*	87.00 ± 1.61***	0.01 ± 0.01	15.91 ± 10.23	72.43 ± 4.33***	0.01 ± 0.01
	*E. faecalis*	N.D.	N.D.	4.65 ± 5.31	N.D.	N.D.	3.13 ± 2.63	N.D.	N.D.	0.39 ± 0.004
H1	*G. adiacens*	37.77 ± 7.64*	26.27 ± 5.28*	0.05 ± 0.02	54.40 ± 3.75*	32.43 ± 4.41	0.08 ± 0.05	37.67 ± 2.85***	25.97 ± 2.21	0.40 ± 0.50
	*K. oxytoca*	6.95 ± 1.32	38.07 ± 2.11****	0.003 ± 0.002	44.07 ± 3.56**	54.37 ± 5.60**	0.01 ± 0.001	25.37 ± 7.01*	68.30 ± 3.80***	0.004 ± 0.004
	*C. freundii*	2.85 ± 0.36	8.93 ± 1.14	0.02 ± 0.01	37.30 ± 1.93*	17.43 ± 2.43	0.02 ± 0.01	9.15 ± 2.92	43.97 ± 10.15*	0.05 ± 0.003
H2	*E. cloacae*	N.D.	N.D.	13.55 ± 2.08	N.D.	N.D.	2.05 ± 0.11	N.D.	N.D.	9.28 ± 0.69
	*S. anginosus* (milleri) group	3.49 ± 0.83**	12.87 ± 1.60*	1.55 ± 0.42	14.37 ± 3.16	25.40 ± 1.14	0.21 ± 0.06	1.59 ± 0.27	13.17 ± 3.96	0.29 ± 0.02
	*E. faecium*	3.35 ± 1.73*	11.32 ± 2.59	6.32 ± 0.57	13.57 ± 1.55	31.97 ± 1.99	8.08 ± 2.19	1.98 ± 0.03	10.88 ± 3.16	1.95 ± 0.14
C1	*E. faecalis*	52.77 ± 27.33	8.97 ± 1.56	0.54 ± 0.06	69.80 ± 4.06*	31.67 ± 10.53	1.10 ± 0.75	65.37 ± 10.17*	35.70 ± 8.51	0.20 ± 0.03
	*S. maltophilia*	15.07 ± 1.72*	9.00 ± 0.76*	0.59 ± 0.07	25.40 ± 6.42	18.43 ± 2.23	0.73 ± 0.15	10.30 ± 1.13	19.30 ± 7.72	1.16 ± 0.26
C2	*E. cloacae*	50.37 ± 6.11**	55.63 ± 4.73***	1.56 ± 0.28	29.87 ± 1.45**	57.03 ± 3.10**	0.13 ± 0.13	80.20 ± 5.55**	51.03 ± 4.36*	0.26 ± 0.09
	*K. pneumoniae*	49.50 ± 9.34*	28.73 ± 0.74**	3.63 ± 0.17	23.03 ± 3.69**	54.53 ± 1.06**	0.46 ± 0.16	53.87 ± 4.78**	39.73 ± 5.42*	0.23 ± 0.04
	*S. anginosus* (milleri) group	7.50 ± 0.98	17.10 ± 4.89	0.11 ± 0.04	34.17 ± 3.96	33.33 ± 4.89	0.27 ± 0.15	9.25 ± 0.45	25.90 ± 1.87	0.18 ± 0.06
	Uninfected	11.41 ± 8.06	9.69 ± 3.37		36.28 ± 6.98	23.31 ± 5.33		15.79 ± 13.22	16.72 ± 8.92	

Results are shown as mean ± SD. Statistical significance was determined using unpaired t-test with Welch’s correction. Results significantly different than the uninfected control are listed in bold (*p < 0.05, **p < 0.01, ***p < 0.001, ****p < 0.0001).

### Bacteria induce pancreatic cell damage including DNA repair response and cell death, which is preventable by antibiotic treatment

**Figure 1.**
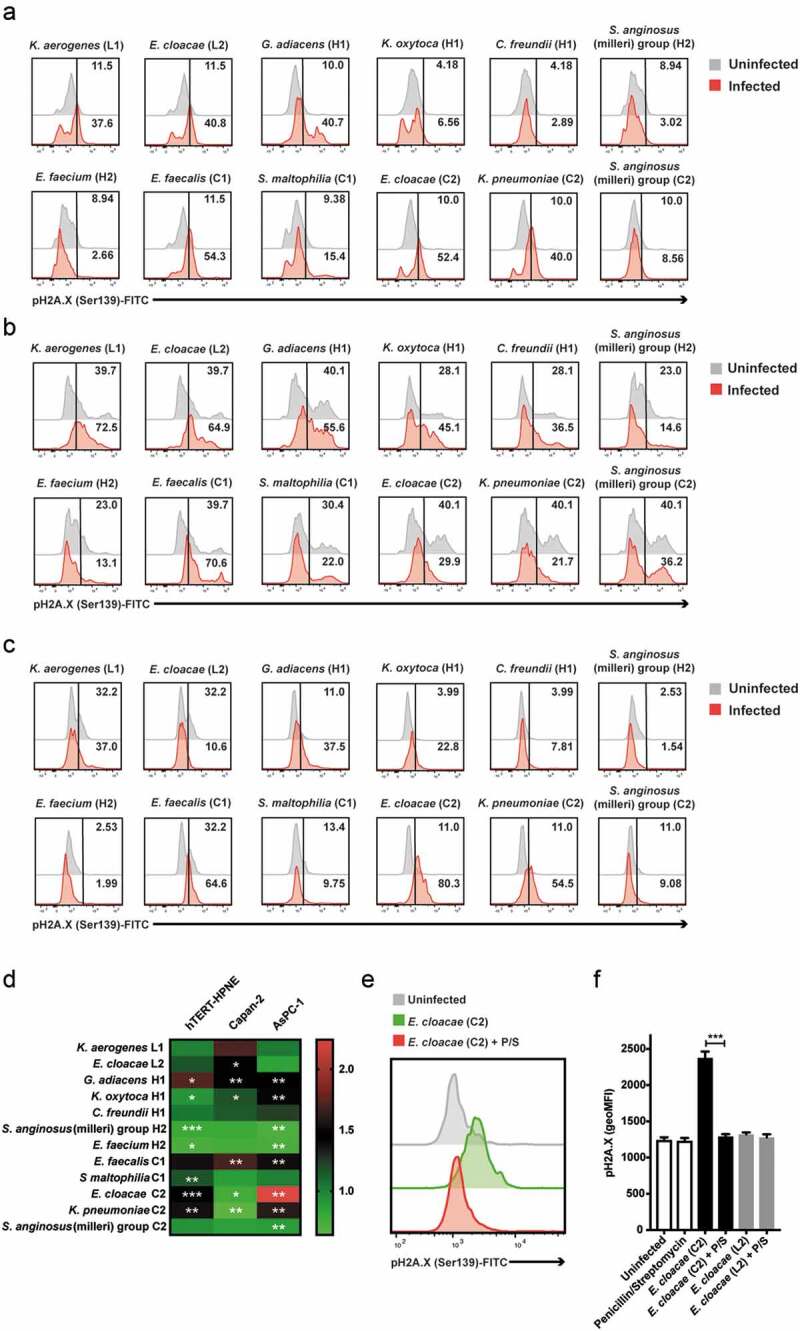
DNA damage induced by infection with bacterial isolates in healthy (hTERT-HPNE), early (Capan-2) and late differentiation stage cancer (AsPC-1) pancreatic cell lines. (a-c) Representative stain of histone H2A.X phosphorylation in response to the isolate panel in hTERT-HPNE (a), Capan-2 (b) and AsPC-1 (c) cell lines. Numbers denote percentage positive events. (d) Histone H2A.X phosphorylation in response to the isolate panel in hTERT-HPNE, Capan-2 and AsPC-1 cell lines (*n* = 3). (e) Representative stain of histone H2A.X phosphorylation in response to *E. cloacae* (C2) with or without penicillin/streptomycin presence in the AsPC-1 cell line. (f) Inhibition of histone H2A.X phosphorylation in response to *E. cloacae* (C2/L2) in the presence of penicillin/streptomycin in the AsPC-1 cell line (*n* = 3). Statistical significance was determined using unpaired t-test with Welch’s correction. Statistical data for pH2A.X relative change (D) was computed on raw geometric MFI values (*p < .05, **p < .01, ***p < .001)

Next, we examined pancreatic cell damage by measuring phosphorylated γH2A.X (pH2A.X), a known hallmark of DNA double strand-breakage and DNA damage response activation, and the extent of cell death after an overnight bacteria co-culture. We found that in nonmalignant pancreatic cells, *Granulicatella adiacens* H1, *K. pneumoniae* C2 and *E. cloacae* C2 strains caused not only significant cell death but also greatest phosphorylation of γH2A.X in the live cell population (Table 3, Figure 1a, d). Similarly, *G. adiacens* H1, *E. faecalis* C1 and *Klebsiella oxytoca* H1 strains also caused strong pH2A.X increase and cell death in both Capan-2 and AsPC-1 (malignant) cell lines (Table 3, Figure 1b-d), while *Klebsiella aerogenes* L1 caused mostly cell death, especially on Capan-2 cells (Table 3). Unlike those strains, *Streptococcus anginosus* (milleri) group (H2/C2) strains, as well as *E. faecium* H2 appeared to spare pancreatic cells from heavy damage, as noted in all three cell lines. Overall, the strongest pH2A.X inducer was *E. cloacae* C2 isolated from an IPMN-Cancer case when co-cultured with AsPC-1 cells, but this effect was fully preventable by applying penicillin-streptomycin in the beginning of bacteria and cell co-culture (Figure 1e, f). Collectively, our data indicate that IPMN cyst-derived bacteria are capable of causing significant DNA damage in pancreatic cells of healthy to early and late cancerous stage. Moreover, Gammaproteobacteria species were among the greatest pH2A.X inducers but antibiotic treatment may prevent the bacteria-induced cellular insult.

## Discussion

The outcome of a pancreatic cancer diagnosis is dismal and the reason PCN, and in particular IPMNs, may undergo cancer transformation remains elusive. Here, we identified culturable members of the pancreatic microbiota found in PCNs, which could be isolated by culturing cyst fluid samples retrieved peri-operatively from PCN patients undergoing pancreatic surgery. We found that bacterial culture-positive cases originated mainly from pancreas cysts histologically classified as IPMNs, particularly in those showing malignant transformations. Other significant factors included patient age, previous history of invasive endoscopy treatment and altered CRP and albumin levels. The finding is in line with a previous study by our group,^11^ showing that cyst fluid from IPMN cases with HGD or invasive cancer and with a history of invasive endoscopic procedures also contains higher levels of bacterial 16S DNA copies. In line with our previous study, which is based on DNA sequencing, the current study now provides evidence that the DNA sequences come from live bacteria, several of the taxa we reported previously were recovered as live bacteria showing functional ability to invade host cells. Importantly, it provides first biological evidence that while some members of pancreatic microbiota appear pose limited effect on pancreatic cells, some including Gammaproteobacteria are capable of inducing cellular damage by causing double-stranded DNA breakage. The latter is a known early step to cell transformation, which appears preventable by an antibiotic pretreatment as shown in our co-culture model. Clinical translation of this information could contribute to advancing the management of PCN patient who are at risk of pancreatic cancer. The origin of bacteria in the pancreas is presently not known. Possible sources include microbial translocation through leaky gut or iatrogenic routes in view of the age and medical history of this patient group.^9,11^ Given that the pancreatic microbiome may promote immune suppression and oncogenesis as recent studies in mice and humans indicate,^8,9,11,14^ improved strategies to prevent bacterial invasion of PCNs are important.

An interesting factor is that all patients in the current study had been given antibiotics prophylaxis before the operation, as the standard of care program. Given that viable microbiota can still be found in the pancreas, the antibiotic prophylaxis effect is restricted. In animal models, as Geller et al. recently reported, the administration of ciprofloxacin intraperitoneally, daily for 6 days could abrogate the pathogenic effect of intratumoral Gammaproteobacteria,^8,10^ which could be a more effective alternative. Those data support the enrichment of Gammaproteobacteria DNA in human pancreatic ductal adenocarcinoma tissues, and instrumentation as a correlative factor to pancreatic microbiome. Consistent earlier findings in pancreatic cyst fluid,^11^ patients who underwent invasive endoscopy treatment also had more culturable Gammaproteobacteria in cyst fluid than those who did not. Given the accumulating evidence that members of the local microbiome may promote progression of PDAC, modulating chemoresistance to adjuvant gemcitabine and affecting patient survival,^8,25^ antimicrobial interventions through the IPMN surveillance programs could perhaps further reduce cancer risk.

Our experimental data provide the first firm evidence that pancreatic cyst fluid-derived viable bacteria are capable of intra-cellular survival and causing DNA damage in human pancreatic cells *in vitro*. These data are consistent with emerging findings that pancreatic cancer harbors intracellular bacteria, enriched with *Enterococcus, Enterobacter, Klebsiella*, and *Citrobacter* in the cancer cells.^26^ It is interesting that we found these bacteria in cyst fluid of IPMNs, which are known precursors to PC, and that that these bacteria are capable of inducing clear DNA damage in human pancreatic cells *ex vivo*. Notably, phosphorylated histone γH2A.X is a marker for, among others, pks^+^ *Escherichia coli* cancer genotoxins.^27^ Here, we observed upregulated phosphorylation of γH2A.X as early as 24 hours post bacterial exposure in pancreatic cells derived from healthy pancreas (hTERT-HPNE) and from pancreatic carcinoma (Capan-2 and AsPC-1). Cellular vulnerability does vary between bacterial strains, and interestingly even between *E. cloacae* strains isolated from cancer associated vs. low-grade IPMN. Unfortunately, it is not possible to know if it is an effect from adaptation to the tumor environment or from specific virulence factors. Consequences of intrapancreatic bacteria reservoir include immune evasion, competition of tissue-resident effector cytotoxic lymphocytes, or immune confusion by altered chemokine induction, which could negatively influence the tumor environment and disease progression. On the other hand, we cannot exclude the possibility of using tumor-associated bacteria as neo-antigens for tumor immunotherapy.

So far, pancreatic cyst fluid-based microbiota studies are scarce, but one study investigated EUS-acquired pancreatic fluid from pancreatitis cases,^28^ showing bacterial culture positivity of 59%. In that study, risk factors included acute pancreatitis and fever, which were not noted in the culture-positive cases here. Through MALDI-TOF analysis, we also observed polymicrobial cultures from PCN fluid included species from the *Enterococcus, Enterobacter, Klebsiella*, and *Citrobacter* genus. It is worth noting that many of the intracystic isolates identified here have also been often found in oral microbiome, with exception of *Citrobacter freundii, Stenotrophomonas maltophilia*, and *K. aerogenes*. Specifically, *S. maltophilia* is known to cause nosocomial infections in the bloodstream, urinary and respiratory tract.^29–31^ Similarly, certain strains of *Enterococcus faecalis* are linked to pancreatitis and cancer,^15^ also are attributable to nosocomial oral infections, specifically in the root canal.^32,33^ Other bacteria linked to cancer include *K. oxytoca*, which was proved to increase in cancer cachexia cases,^16^ and *K. pneumoniae*, which is associated with the development of colorectal cancer in patients with pyogenic liver abscess.^24^ *Klebsiella* species have been described as extremely starvation tolerant in other mucin-rich environments.^34^ *G. adiacens*, intriguingly, has been proposed as a cancer biomarker, including pancreatic cancer,^11,17,18^ lung cancer,^19,20^ and oral squamous cell carcinoma.^21^ *S. anginosus* is connected to colorectal cancer^22^ and head and neck squamous cell carcinoma.^23,35^

In conclusion, this study provides a first in-depth report of the culturable PCN microbiota with new insights to the unresolved link of PCN to cancer. The strength of this study is that all samples were acquired from operating theaters in sterile condition and not by endoscopy, handled by a clinical laboratory certified with “ISO 9001:2015 standard” GLP quality control, the risk of contamination (gut microbiota or environmental) or extra-pancreatic infection is hence minimal. Some limitations include the non-cultivable part of the pancreatic microbiome and lack of bacterial genetic analysis. A larger prospective study is needed to confirm the frequency of culture positive PCN cases, and permit data integration for multivariate analysis as well as to identify demographics and clinical confounders. Future studies tackling these challenges shall provide further insights into the functional role of the pancreatic microbiome in the progression from healthy pancreas to cancer. In the context of PCN/IPMN, this may provide a window of opportunity for cancer prevention. Targeted administration of antimicrobial agents by, for example, endoscopy-assisted delivery remains to be tested to reduce bacterial risks in invasive endoscopy procedures.

## Materials and methods

### Cohort description

Cyst fluid samples analyzed in this study were collected from 29 patients undergoing pancreatic surgery due to PCN with suspected malignancy, with a diagnosis of pancreatic cystic lesions based on preoperative diagnosis at the Karolinska University Hospital Huddinge, Sweden. Each patient signed an informed consent form prior to the collection of cyst fluid. This study follows the Helsinki convention and good clinical practice and was approved by the Regional Ethics Committee of Stockholm (Dnr. 2015/1580-31/1). Clinical and laboratory data were extracted from electronic journals by clinical doctors. Patients were subgroups according to their histopathology diagnosis of the pancreas tissues.

### Cyst fluid collection and bacterial culture

One to five mL of cyst fluid was aspirated in surgery theater under sterile condition from pancreatic cysts with sterile syringes immediately after the surgical resection, and immediately injected into an anaerobic BacT/ALERT® FN Plus (bioMérieux, Marcy-l’Étoile, France) blood culture bottle. In case the lower limit of 1 mL was not reached, fluid from several cysts was pooled together. All study material was provided by Karolinska University Hospital´s Clinical Microbiology Laboratory, a certified “ISO 9001:2015 standard” laboratory that provides the GLP quality standard. Both study materials and all steps were screened regularly to ensure they are contamination free; this includes blank sampling tubes, and tools during sampling and culturing. Additionally, all our samples were handled inside biosafety cabinet class II. Sample bottles were then incubated in the BacT/ALERT 3D (Bio-Merieux, France) system until they signaled for positivity for a maximum of 5 days. In positive culture bottles, samples were Gram stained and then subcultured on agar plates. Colonies that grew on agar were subjected to species identification by matrix-assisted laser desorption/ionization time-of-flight mass spectrometry (MALDI-TOF MS). As for the MALDI-TOF MS mass spectrometry, pure colonies obtained were identified following culture with the MALDI-TOF MS Biotyper® System (Bruker Daltonik, Bremen, Germany). Samples were spotted on steel MALDI-TOF MS target plates in duplicates, and 1 μl of α-Cyano-4-hydroxycinnamic acid (HCCA) Matrix was added to each sample spot. MALDI-TOF MS microflex LT/SH System along with the software Bruker Biotyper 3.1 (version 4613). MALDI-TOF MS scores ≥1.70 and ≥2.00 were accepted as successful identifications at genus and species level, respectively, as recommended in criteria for data interpretation set by the manufacturer.

### Pancreatic cell lines

Pancreatic cell lines hTERT-HPNE (ATCC^®^ CRL-4023™), Capan-2 (ATCC^®^ HTB-80™) and AsPC-1 (ATCC^®^ CRL-1682™) were obtained from ATCC (Manassas, VA, USA). Each cell line was maintained in specialized medium according to the supplier’s specifications.

### Bacterial co-cultures

Cell lines were cultured overnight in RPMI-1640 medium (Thermo Fisher Scientific, Waltham, MA, USA) supplemented with 2% human serum obtained from the Blood Transfusion Clinic (Karolinska University Hospital, Huddinge, Sweden). On the day of the experiment, bacterial cultures were diluted in RPMI-1640 + 2% human serum and added to the pancreatic cells at a final multiplicity of infection of 1 CFU/cell. In some co-cultures, penicillin-streptomycin (Thermo Fisher Scientific) was added to a final concentration of 5 mM. The cells and bacteria were then co-cultured at 37°C, 5% CO_2_ for either 2 h (for gentamicin protection assay) or 24 h (for DNA damage and cell death assay).

### Gentamycin protection assay

The bacterial invasiveness was determined as earlier described^36^ by co-culture with each pancreatic cell line. Briefly, after 2 h of incubation at 37°C, 5% CO_2_, the medium was removed from each co-culture well without disturbing the cells adhering to the bottom of the well. The wells were then washed twice with 100 µL PBS prior to the addition of 100 µL of RPMI + 2% HuS medium supplemented with 100 µg/mL gentamicin, to eliminate bacteria not residing intracellularly. After 1 h incubation at 37°C and 5% CO_2_, the gentamicin medium was removed, and the wells washed as mentioned above. The cells were then lysed with 100 µL of 0.1% Triton X-100 and plated both undiluted and in 1:10 dilution on Blood Agar or CHOC plates supplemented with pyroxidal (*Granulicatella adiacens*). After overnight incubation at 37°C, 5% CO_2_, CFU count was determined and expressed as percentage of recovered bacterial cells for each isolate relative to the input bacterial cells.

### DNA damage and cell death assay

After 24 h of co-culture at 37°C, 5% CO_2_, the medium was aspirated from the wells and cell layer detached using trypsin-EDTA (Thermo Fisher Scientific). The cells were washed with FACS buffer (PBS + 2% FCS + 2 mM EDTA) and stained with LIVE/DEAD Fixable Near-IR Dead Cell Stain (Thermo Fisher Scientific) for 20 minutes on ice. After washing away excess reagent with FACS buffer, the cells were stained with H2A.X Phosphorylation Assay Kit for Flow Cytometry (MilliporeSigma, St Louis, MO, USA), in accordance with the manufacturer’s instructions. Stained samples were acquired on a FACSVerse flow cytometer (BD Biosciences, Franklin Lakes, NJ, USA). Single-stained polystyrene beads (BD Biosciences) were used for compensation. Flow cytometry data analysis was performed in FlowJo software v10.6.2 (BD).

## Statistical analysis

Descriptive analyses were performed on clinical characteristics and presented as percentages or mean and standard deviation. Statistical analysis was done using GraphPad Prism Version 7.0 c and 9.0.0. For quantitative data, the unpaired t-test with Welch’s correction or Mann-Whitney U test was used. Datasets also initially underwent normality distribution testing. For nominal datasets, Fisher´s exact test was used using MedCal software calculator. Two-sided *p*-values <0.05 were considered significant.
